# Intraovarian Platelet-Rich Plasma Administration for Anovulatory Infertility: Preliminary Findings of a Prospective Cohort Study

**DOI:** 10.3390/jcm13175292

**Published:** 2024-09-06

**Authors:** Anastasios Potiris, Sofoklis Stavros, Charalampos Voros, Panagiotis Christopoulos, Abraham Pouliakis, Michael Savvidis, Angeliki Papapanagiotou, Theodoros Karampitsakos, Spyridon Topis, Tereza Vrantza, Maria Salvara, Angeliki Gerede, Sophia Anysiadou, Georgios Daskalakis, Peter Drakakis, Ekaterini Domali

**Affiliations:** 1 Third Department of Obstetrics and Gynecology, University General Hospital “ATTIKON”, Medical School, National and Kapodistrian University of Athens, 124 62 Athens, Greece; apotiris@med.uoa.gr (A.P.); sfstavrou@med.uoa.gr (S.S.); theokarampitsakos@hotmail.com (T.K.); spyros.topis1996@gmail.com (S.T.); terezavrantza@hotmail.com (T.V.); pdrakakis@med.uoa.gr (P.D.); 2 First Department of Obstetrics and Gynecology, Alexandra Hospital, Medical School, National and Kapodistrian University of Athens, 115 28 Athens, Greece; charalamposvoros@hotmail.com (C.V.); sofanysi@yahoo.gr (S.A.); gdaskalakis@med.uoa.gr (G.D.); 3 Second Department of Obstetrics and Gynecology, Aretaieion University Hospital, Medical School, National and Kapodistrian University of Athens, 115 28 Athens, Greece; panchrist@med.uoa.gr; 4 Second Department of Pathology, University General Hospital “ATTIKON”, Medical School, National and Kapodistrian University of Athens, 124 62 Athens, Greece; apouliak@med.uoa.gr; 5 Department of Biomedical Sciences, Faculty of Health and Caring Professions, University of West Attica, 122 43 Athens, Greece; na.plias@yahoo.com; 6 Department of Biological Chemistry, Medical School, National and Kapodistrian University of Athens, 115 27 Athens, Greece; agpana@med.uoa.gr; 7 Medical School, National and Kapodistrian University of Athens, 115 27 Athens, Greece; m.salvara@outlook.com; 8 Department of Obstetrics and Gynecology, Democritus University of Thrace, 691 00 Campus, Greece; agerede@otenet.gr

**Keywords:** infertility, platelet-rich plasma (PRP), reproductive hormones, anovulatory cycles, ovarian rejuvenation

## Abstract

**Background/Objectives**: Infertility constitutes a significant challenge for couples around the world. Ovarian dysfunction, a major cause of infertility, can manifest with anovulatory cycles, elevated follicle-stimulating hormone levels, and diminished ovarian reserve markers such as anti-Müllerian hormone (AMH) levels or the Antral Follicle Count (AFC). Blood-derived therapies including platelet-rich plasma (PRP) have been used in fertility treatments in women with low ovarian reserve or premature ovarian insufficiency. This prospective clinical cohort study aims to assess the effects of intraovarian PRP therapy on ovarian function in women diagnosed with anovulatory cycles. **Methods**: The preliminary findings of this prospective cohort study are based on the first 32 patients enrolled. In this study, patients over 40 years old with anovulatory infertility were included. Venous blood samples were collected from each participant for the preparation of autologous platelet-rich plasma (PRP). Each participant received two courses of intraovarian PRP injections using a transvaginal ultrasound-guided approach. Serum levels of reproductive hormones before and after PRP intervention were measured. **Results**: This study’s results demonstrate a significant improvement in ovarian physiology following transvaginal ultrasound-guided PRP infusion. A 75% increase in Antral Follicle Count (AFC) was observed, which was statistically significant. Furthermore, statistically significant reductions in follicle-stimulating hormone (FSH), luteinizing hormone (LH), and prolactin levels were observed. Serum Vitamin D 1–25 levels were substantially increased after the injection. **Conclusions**: These findings highlight the beneficial impact of intraovarian PRP injection in optimizing ovarian function and other metabolic parameters. However, the published literature on this subject is limited and further clinical studies should be conducted to confirm the role of intraovarian PRP in fertility treatments.

## 1. Introduction

According to recent data, infertility is a health disorder affecting 48 million couples and 186 million individuals worldwide [1]. The American College of Obstetricians and Gynecologists (ACOG) describes infertility as the inability to achieve a pregnancy after twelve months of regular unprotected sexual intercourse or therapeutic donor insemination in women under 35 years or after 6 months in women older than 35 years [2]. A significant proportion of infertility cases can be attributed to ovarian disorders, particularly anovulatory cycles, which are characterized by the absence of ovulation [3]. Ovarian dysfunction often manifests as elevated levels of follicle-stimulating hormone (FSH), thereby highlighting the importance of assessing and addressing ovarian function in infertility management [4]. Furthermore, elevated levels of FSH are associated with diminished ovarian reserve, which refers to a woman of reproductive age who has regular menses but reduced fecundity or decreased response to ovarian stimulation compared with age-matched women [5]. Testing for ovarian reserve can be made with either anti-Müllerian hormone (AMH) levels or the Antral Follicle Count (AFC) [6]. Both methods are equal in assessing ovarian reserve according to the European Society of Human Reproduction and Embryology (ESHRE) [7].

Blood-derived therapies involve the local administration of either whole blood (autologous whole blood) or platelets (platelet-rich plasma, PRP) resulting in higher concentrations of cytokines, which promote the growth and differentiation of repair cells [8]. Platelets are found at the site of tissue injury and, once activated, platelets release their intracellular cytoplasmic granules [9]. The platelet granules contain platelet-derived growth factor (PDGF), transforming growth factor (TGF)-beta, fibroblast growth factor (FGF), vascular endothelial growth factor (VEGF), and epidermal growth factor (EGF) [10]. PRP is defined as a platelet-rich concentrate with platelet levels greater than the baseline count in whole blood and is manufactured using differential centrifugation of blood [11]. In recent years, the PRP method has already been used in various fields of medicine, and it is well known for its tissue rejuvenation and angiogenesis properties [12,13].

Moreover, PRP therapy has been used as an adjunct to fertility treatments in women with low ovarian reserve or premature ovarian insufficiency [14]. The recent literature in humans and animals suggests that intraovarian PRP administration in poor ovarian reserve may restore ovarian function and increase the chances of pregnancy [15]. This prospective clinical cohort study aims to assess the effects of intraovarian PRP therapy on ovarian function in women diagnosed with anovulatory cycles. Ovarian function is assessed by changes in the levels of expressed hormones and the Antral Follicle Count. Furthermore, the effects of PRP in liver, kidney, and thyroid parameters are the secondary outcomes of the present study. Ultimately, this study aims to determine the viability of PRP therapy as a therapeutic option for infertile women with anovulatory cycles.

## 2. Materials and Methods

### 2.1. Study Registration and Ethics Approval

This is the first preliminary update on an officially registered prospective cohort study including women who underwent PRP treatment from March 2022 to November 2023 at our clinical and research center at the First Department of Obstetrics and Gynecology of the National and Kapodistrian University of Athens in Alexandra General Hospital. The study received ethics approval from the Ethics Committee and Institutional Research Board of Alexandra Hospital, with registration number 758/19-10-2023. All individuals who participated in the study provided written informed consent regarding study participation and publication of their medical records provided that the confidentiality of their medical information is preserved.

### 2.2. Sample Size Calculation

A power analysis was performed to assess the appropriate study sample and ensure sufficient statistical power of the study. Thorough research of the existing literature and subsequent analysis of the relevant studies was conducted to assess the effect size in our power analysis. A moderate effect size (Cohen’s d = 0.5) for the primary outcome variables, reproductive hormone levels, was based on this literature review and expert input. A statistical power level of 80% (1 − β = 0.80) was chosen to enhance the likelihood of detecting true effects. The significance level (α) was set at 0.05, representing a 5% risk of a Type I error. We planned to employ paired t-tests, non-parametric tests for paired data, and the Wilcoxon signed rank test to compare the means of measurements. Considering the association between pre- and post-intervention measurements, a moderate correlation coefficient of 0.5 was assumed based on similar studies. Utilizing these parameters, we used statistical software G*Power 3.1 [16] to perform the power analysis. Power analysis calculated a sample size of 72 participants for the predetermined statistical power. Practical considerations, including patient availability and resource constraints, were factored in to ensure the study’s feasibility within the clinical setting.

### 2.3. Inclusion and Exclusion Criteria

This is the first preliminary update of the prospective cohort study results including the first 32 patients enrolled. Our inclusion criteria included women who were ≥40 years old and were diagnosed with infertility due to anovulatory menstrual cycles. Individuals with coexisting medical conditions that could confound the study outcomes were excluded. These conditions included polycystic ovary syndrome (PCOS), thyroid disorders, diabetes mellitus, adrenal disorders, sexually transmitted diseases, chronic inflammatory diseases, endometriosis, chronic endometritis, a Body Mass Index (BMI) above 35 or less than 18.5, and hypothalamic–pituitary disorders. Moreover, participants with a history of prior PRP therapy for any medical condition were excluded to ensure that the effects of PRP were assessed independently in this study. Finally, individuals unable to provide informed consent due to cognitive impairments or language barriers were not considered for participation.

### 2.4. Preparation of PRP Solution

Venous blood samples were collected from each participant under aseptic techniques to prevent contamination and maintain the sterility of the collected blood. A sufficient blood volume, typically 65 to 70 milliliters, was obtained. The tubes contained an anticoagulant factor, typically citrate dextrose 1.5 mL, in each vial. ACD-A Anticoagulant Citrate Dextrose Solution, Solution A, USP (2.13% free citrate ion), is a sterile, non-pyrogenic solution. ACD-A is approved by the United States Food and Drug Administration (FDA) for use in autologous PRP [11]. Blood samples were subjected to a controlled centrifugation process using a high-quality centrifuge precisely calibrated for PRP preparation. Two centrifugations were performed in a digital refrigerated centrifuge at carefully selected parameters. Due to the first centrifugation, the speed was 800 revolutions per minute (RPM) in 148g, the duration was 10 min, and the temperature was 20 °C. The first centrifugation separated the blood components into distinct layers, resulting in platelet-poor plasma (PPP) at the top, PRP in the middle, the buffy coat, and red blood cells (RBCs) at the bottom. The PRP layer is characterized by its golden-yellow appearance. We precisely extracted the PRP-PPP layers and underwent a second centrifugation step to further concentrate platelets and growth factors [17]. The centrifugation parameters for the second centrifugation were 800 (RPM) in 370 g for 10 min at 20 °C. The second centrifugation further separated the PRP layer, yielding a highly concentrated PRP solution with enhanced regenerative potential. More specifically, the top 2/3 of the serum were extracted, which contained plasma-poor platelets (PPP). The remaining layer was the PRP layer. The highly concentrated PRP solution underwent volume adjustments to reach the desired platelet concentration. The initial concentration of platelets was approximately 200,000–300,000 platelets/μL. The goal concentration of platelets in PRP was approximately 1,000,000 platelets/μL and the typical range of PRP concentration ranged from 650,000 to 750,000 platelets/μL. PRP sterile vials or syringes were chosen for serum preservation. PRP intraovarian infusion was performed immediately following preparation. Each container was appropriately labeled with participant identifiers to prevent any potential mix-up or confusion.

### 2.5. Transvaginal Ultrasound-Guided PRP Injection Process

Each participant underwent an intraovarian transvaginal ultrasound-guided PRP injection. The intervention was held at Alexandra Hospital. Left and right ovaries were visualized, and the PRP solution was injected intramedullary across the central part of each ovary, using a 17-gauge single-lumen needle. The ultrasound-guided method was chosen to ensure the accurate targeting of the ovaries. Participants received two courses of PRP treatments during the early follicular phase of the cycle over a 4-month period. PRP solution was prepared on administration day immediately after blood sample collection. The intervals between injections were standardized to optimize the therapeutic effect. Before the first application of PRP, hormonal profiles of FSH, LH, E2, TESTO, 17-OH-Progesterone, prolactin, and cortisol on days 2–4 were measured. Menstrual cycle, documentation of menstrual period patterns, and patients’ history were documented. Participants were closely monitored throughout the study. Regular follow-up visits allowed for the continuous assessment of changes in hormone levels. Blood samples were collected on days 2 to 4 of the next two consecutive menstrual periods.

### 2.6. Statistical Analysis

Statistical analysis was performed via the SAS for Windows 9.4 software platform (SAS Institute Inc., Cary, NC, USA). Descriptive values were expressed as median and quartile 1 to quartile 3 range (Q1–Q3). For completeness reasons using the mean ± standard deviation (SD), descriptive statistics for categorical data are expressed in frequencies and percentages. Normality was not always ensured via the Shapiro–Wilk test; thus, comparisons between the time points were performed using the paired Wilcoxon signed rank test. The significance level (*p*-value) was set to 0.05.

## 3. Results

Out of the 32 patients, 11 (34.4%) were smokers, and 14 (43.8%) reported alcohol consumption. The median age was 44 years (Q1–Q3: 41.5–45.5 years, min: 40, max: 50), and the median BMI was 28 Kgr/m^2^ (Q1–Q3: 26–30 Kgr/m^2^, min: 22, max: 33). Data regarding biomedical markers have been organized into different time points: the menstrual cycle before the PRP injection procedure (before PRP), earlier on the day of the first PRP injection procedure (during first PRP), the menstrual cycle between the two PRP injection procedures (between PRPs), earlier on the day of the second PRP injection procedure (during second PRP), one and two months after the second PRP injection procedure (check 1 and check 2, respectively). Table 1 represents the measured values for each time point.

Subsequently, we evaluated if there was an increasing or decreasing trend in the study biomarkers by fitting a line for each measure during the time points and evaluating the slope (a negative slope indicates a decreasing trend and a positive slope indicates an increasing trend). Different lines were fitted for each patient. The results are presented as mean value and SD of the slopes (Table 2); for the complete period, there is a reduction in FSH, LH, E2, progesterone, prolactin, testosterone, HDL, and Trg and increases in Vitamin D, B12, γGT, and progesterone; for TSH and T3 there are very small values of the slope, leading towards a decision that TSH and T3 remain unchanged.

Furthermore, we applied paired tests to evaluate differences (a.) before PRP and between the two PRPs and (b.) between PRPs and after the second PRP (one month after the second PRP). The observed changes and relevant correlations of the most significant measured values at different time points are presented in Table 3. The complete study results are presented in Supplementary Table S1 (Table S1). The Antral Follicle Count (AFC) increased by 75% in individuals before the intraovarian infusion and by 71.9% between PRP treatments and after the first and second menstrual periods, respectively. Additionally, we observed a statistically significant decrease in FSH before injection, with a mean value of 17.9 mIU/m reducing to 9.06 at the first check and further to 8.38 mIU/m at the second check, all with a *p*-value < 0.05. The serum level of luteinizing hormone (LH) also decreased, measuring 15.15 mIU/m before the intervention, 6.66 mIU/m at the first check, and 6.88 mIU/m at the second check, all with a *p*-value < 0.05. Furthermore, a statistically significant increase in Vitamin D 1–25 serum levels was noted between two PRP injections and at the first and second checks, progressing from 35.8 mg/dL to 41.65 mg/dL and 41.6 mg/dL, respectively. Prolactin levels decreased before PRP and at the first and second checks, with mean values of 8.23 mIU/m, 6.85 mIU/m, and 6.43 mIU/m, respectively. Cholesterol levels showed a statistically significant decrease before PRP injection and after the first menstrual period. Similarly, triglyceride serum levels decreased before PRP and after the first and second menstrual periods, declining from 81 mg/dL to 73.5 mg/dL and 68 mg/dL, respectively. Moreover, we identified a statistically significant increase in γ-glutamyltransferase (γ-GT) (*p* < 0.05), rising from 18 IU/mL to 22 IU/mL and 25 IU/mL. Creatinine serum levels decreased from the first to the second menstrual period after the PRP intraovarian infusion.

## 4. Discussion

Until now, there has not been a specific treatment for women suffering from infertility associated with anovulatory menstrual periods due to poor ovarian reserve. Platelets carry over 800 protein molecules, cytokines, hormones, and growth factors [11]. When activated, platelets release many biologically active proteins that stimulate cell multiplication, growth, and differentiation [18]. PRP derives from whole blood by centrifugation and separation of the components [12]. In this way, red cells are removed, and the growth factors included in the plasma are increased. It promotes the release of growth factors such as the insulin-like growth factor-1 (IGF-1), platelet-derived growth factor (PDGF), transforming growth factor-beta 1 (TGF-β1), and vascular endothelial growth factor (VEGF). According to the literature, those factors promote soft tissue healing, tissue regeneration, angiogenesis, osteogenesis, and reduce inflammation and infection [19,20]. Furthermore, platelets release several cytokines which play a significant role in follicle development. Hence, it should be investigated if PRP acts as a stimulus in the ovaries, promoting oogenesis and follicular growth [21].

Our research reveals novel outcomes regarding the intraovarian injection of platelet-rich plasma (PRP). Notably, the Antral Follicle Count (AFC) significantly increased, indicating a promising response to the intraovarian PRP treatment, and holds significant implications for fertility preservation and reproductive health. Moreover, the present study has shown important alterations in the hormonal profiles of individuals following PRP intervention. More specifically, a significant decrease in follicle-stimulating hormone (FSH) levels was evident post-PRP infusion. Similarly, luteinizing hormone (LH) levels declined after the second infusion, which is indicative of a potential modulation in the hypothalamic–pituitary–ovarian axis. These hormonal changes suggest a potential improvement in ovarian function. We expect our results to be more trustworthy with a larger sample size. We also showed a notable increase in Vitamin D 1–25 serum levels post-PRP treatment, suggesting a potential role in ovarian health. Furthermore, alterations in liver function markers were observed, with a surge in γ-glutamyltransferase (γ-GT) levels after the intervention. These results highlight the need for further investigation into the systemic effects of PRP therapy beyond ovarian function.

Anovulatory cycles due to poor ovarian reserve (POR) are one of the main etiologies of infertility in women of advanced maternal age [22]. The most sensitive and commonly used marker to assess ovarian reserve is FSH. Intraovarian injection with PRP therapy seems promising for these patients. Cakiroglu et al. performed the first cohort study in 2020, including 311 patients with POI, showing no significant increase in FSH levels and an increase in AMH levels and AFC after this intervention; however, only the FSH finding was clinically significant (*p* < 0.01) [23]. On the other hand, Melo et al. detected that the group of patients with PRP infusion had statistically significant improvements in serum levels of FSH, AMH, and AFC. In contrast, there was no change in the control group [24].

In a recent study, Sills et al. delved into the potential impacts of administering autologous platelet-rich plasma (PRP) through intraovarian injections to foster ovarian rejuvenation. The study enlisted 50 participants between 27 and 40, all expected to exhibit a diminished response to ovarian stimulation and classified under POSEIDON 3 or 4. Furthermore, 64% of these individuals had a history of ovarian retrieval [25]. Farimani et al. in 2021 and Navali et al. in 2022 highlighted the positive outcomes observed in women with reduced ovarian reserve or premature ovarian failure following intraovarian PRP therapy. These improvements encompass increased antral follicle count, hormone profile, and successful pregnancy rates [26,27]. Our investigation aligns with these reports due to changes in reproductive hormones and AFC, providing further support for the beneficial effects of PRP on ovarian function. Reduced levels of follicle-stimulating hormone (FSH) and an increase in both the quantity of oocytes and the number of mature oocytes were observed.

In the study by Cakiroglu et al., they followed 311 women who had previously been diagnosed with primary ovarian insufficiency with intraovarian PRP injections. Twenty-three were able to achieve spontaneous pregnancy (7.4%), and eight-two (26.3%) developed at least one cleavage-stage embryo after undergoing ovarian hyperstimulation for IVF [23]. There was also an increase in AMH following intraovarian PRP treatment (0.18 ± 0.18 ng/mL vs. 0.13 ± 0.16; *p* < 0.01); however, FSH was not statistically significantly different (41.6 ± 24.7 vs. 41.9 ± 24.7, *p* = 0.87) [23]. Two years later, the same authors published a study with 510 women, and PRP treatment presented favorable results, leading to an improvement in AFC, higher serum AMH levels, decreased serum FSH levels, a higher number of mature oocytes, and blastocyst-stage embryos. In our study, even with fewer patients, we detected improvement in FSH and AFC. Following PRP injection, spontaneous conception occurred in 22 women (4.3%) and 474 women (92.9%) pursued in vitro fertilization (IVF). Among those attempting IVF, 65.8% achieved oocyte fertilization and underwent embryo transfer, 17.5% had an ongoing pregnancy, and 11.4% had a live birth [28]. In a recent study with 60 women between 30 and 42 years of age treated by PRP injection in the ovaries, an increase in retrieved oocytes but no significant changes in euploid blastocyst development or pregnancy rates between the two study groups was observed [14].

The fact that the presented results are part of the preliminary update of the cohort is a potential limitation of the present study. Furthermore, participation in this study was voluntary and thus will constitute a selection bias of the present study. The methodical approach, preparation, and administration of PRP guided by ultrasound are strengths of the study. Moreover, the fact that additional parameters were measured assessing the systematic effects of PRP is also a strength of this study.

## 5. Conclusions

The preliminary results of an ongoing cohort study on transvaginal ultrasound-guided platelet-rich plasma (PRP) infusion into the ovaries revealed significant Antral Follicle Count (AFC) increases, indicating enhanced follicular development. Concurrent statistically significant decreases in follicle-stimulating hormone (FSH), luteinizing hormone (LH), and prolactin levels suggest hormonal modulation post-PRP infusion. Furthermore, elevated Vitamin D 1–25 levels and decreased cholesterol/triglyceride levels imply metabolic improvements. Increased γ-glutamyltransferase (γ-GT) levels and decreased creatinine levels imply systematic effects of PRP administration. These findings highlight the beneficial impact of intraovarian PRP injection in optimizing ovarian function and metabolic parameters. However, the published literature on this subject is limited and further results after completion of the cohort are necessary to confirm the beneficial effect of PRP intraovarian treatment.

## Figures and Tables

**Table 1:** Median and Q1–Q3 range of the measured values for each time point.

Measured Value	Before PRP	During 1st PRP	Between PRPs	During 2nd PRP	Check 1	Check 2
AFC	3 (2–5)		3 (2–4)		4 (3–4.5)	5 (3–7.5)
FSH	17.9 (13.15–22.15)	9.13 (7.59–18.55)	12.95 (9.37–15.1)	12.62 (8.68–15)	9.06 (7.58–12.4)	8.38 (7.75–10.6)
LH	15.15 (12.85–18.1)	10.5 (6.73–19.25)	8.08 (4.35–13.2)	9.47 (7.53–14.7)	6.66 (4.85–10.75)	6.88 (5.1–8.9)
E2	44 (30.92–96.9)	67 (45–198.68)	71.25 (45.3–138.79)	129 (73.5–189)	73.05 (50.27–109)	89 (53–110.16)
Progesterone	0.97 (0.74–1.23)	1.1 (0.72–2.22)	1.1 (0.56–1.67)	1.16 (0.66–1.99)	0.89 (0.68–2.2)	0.78 (0.65–1.65)
Vitamin D 1.25	40 (33.1–47.2)	35.2 (27.3–49)	35.8 (28.6–42.5)	37.13 (29.9–45.35)	41.65 (35–46.85)	41 (33.6–53.2)
Vitamin B12	430.5 (391.5–457.5)	445.5 (388.5–503)	415.5 (385–467)	440.5 (389–463.5)	450.5 (379–505)	466 (415.5–513.5)
Prolactin	8.23 (5.25–11.3)	8 (5.28–12.5)	7.77 (6.38–11)	7.74 (5.6–11.1)	6.85 (5.4–9.68)	6.43 (4.5–8.74)
Testosterone	23.1 (19.25–32.3)	27.65 (20.82–32.56)	27.58 (20.99–31.2)	26.9 (21.38–32.84)	23.57 (18.5–30.1)	23.3 (20.19–32.1)
Cortisone	13.65 (8.9–26.8)	12.2 (8.6–22.04)	10.15 (6.78–18.05)	11.4 (9.42–17.44)	11.17 (9.4–21)	11.3 (8.9–14.45)
Cholesterol	166 (132–201)	157.5 (125–179)	166.5 (156–194)	172 (143–199)	142.5 (107–156)	133 (100.5–169.5)
HDL	60.5 (53.5–71)	59.5 (55–67.5)	65.5 (57.5–76.5)	68 (58–77.5)	66 (57.5–72.5)	60 (48.5–69)
Trg	81 (67–105.5)	87.5 (67.5–116)	88.5 (50–100)	79.5 (58.5–105)	73.5 (45.5–107.5)	68 (57.5–88)
γGT	18 (13.5–21.5)	20 (14.5–29)	17.5 (13.5–26)	20 (16–27)	22 (17–30)	25 (21–29.5)
Creatinine	0.73 (0.58–0.99)	0.78 (0.65–1.15)	0.83 (0.56–1.16)	0.78 (0.61–1.05)	0.88 (0.63–0.99)	0.65 (0.55–0.78)
Bilirubin total	0.53 (0.4–0.61)	0.44 (0.33–0.56)	0.5 (0.4–0.67)	0.6 (0.4–0.7)	0.4 (0.3–0.75)	0.4 (0.33–0.5)
Bilirubin direct	0.1 (0.1–0.2)	0.2 (0.1–0.2)	0.1 (0.1–0.2)	0.2 (0.1–0.3)	0.1 (0.1–0.2)	0.1 (0.1–0.2)
Progesterone	0.69 (0.53–1.22)	0.79 (0.68–1.58)	0.72 (0.61–1.5)	0.88 (0.65–1.69)	0.71 (0.53–1.4)	0.74 (0.55–1.34)
TSH	1.58 (1.05–2.1)	1.93 (1.35–2.25)	1.7 (1.12–2.2)	1.57 (1.37–2.34)	1.77 (1.56–1.9)	1.99 (1.65–2.15)
T3	1.5 (1.21–1.62)	1.22 (1–1.56)	1.62 (1.23–2)	1.77 (1.21–2.06)	1.65 (1.45–2.05)	1.44 (1.21–1.77)

**Table 2:** Mean value and standard deviation of slope for the studied measures for the complete time frame of the study (i.e., up to two months after the second PRP).

Slope	Mean ± SD
FSH	−0.12 ± 0.21
LH	−0.08 ± 0.08
E2	−0.55 ± 1.38
Progesterone	−0.01 ± 0.07
Vitamin D	0.08 ± 0.18
Prolactin	−0.05 ± 0.06
Vitamin B12	0.26 ± 1.37
Testosterone	−0.01 ± 0.11
HDL	−0.03 ± 0.16
Trg	−0.24 ± 0.38
γGT	0.04 ± 0.17
Progesterone	0.01 ± 0.03
TSH	0 ± 0.01
T3	0 ± 0.01
FT4	0 ± 0.01

**Table 3:** The number and percentage of cases according to changes in different time points and *p*-values of the same correlations.

Measured Value	Changes before PRP and between PRPs	Changes between PRPs and Check 1	Changes before PRP and Check 1	Changes before PRP and Check 2	Changes between PRPs and Check 2	Changes between Check 1 and Check 2	*p*-Value before PRP and between the PRPs	*p*-Value between PRPs and Check 1	*p*-Value before PRP and Check 1	*p*-Value before PRP and Check 2	*p*-Value between PRPs and Check 2	*p*-Value Check 1 and Check 2
AFC	increase: 7 (21.9%) decrease: 14 (43.8%) same: 11 (34.4%)	increase: 14 (43.8%) decrease: 7 (21.9%) same: 11 (34.4%)	increase: 11 (34.4%) decrease: 16 (50%) same: 5 (15.6%)	increase: 24 (75%) decrease: 3 (9.4%) same: 5 (15.6%)	increase: 23 (71.9%) decrease: 4 (12.5%) same: 5 (15.6%)	increase: 23 (71.9%) decrease 6 (18.8%) same: 3 (9.4%)	0.192	**0.019**	0.329	**<0.001**	**<0.001**	**<0.001**
FSH	increase: 7 (21.9%) decrease: 25 (78.1%)	increase: 9 (28.1%), decrease: 23 (71.9%)	increase: 1 (3.1%) decrease: 31 (96.9%)	increase: 2 (6.3%) decrease: 30 (93.8%)	increase: 6 (18.8%), decrease: 26 (81.3%)	increase: 14 (43.8%) decrease: 18 (56.3%)	**<0.001**	**0.005**	**<0.001**	**<0.001**	**<0.001**	0.233
LH	increase: 6 (18.8%), decrease: 26 (81.3%)	increase: 15 (46.9%) decrease: 17 (53.1%)	increase: 3 (9.4%) decrease: 29 (90.6%)	increase: 4 (12.5%) decrease: 28 (87.5%)	increase: 14 (43.8%) decrease: 18 (56.3%)	increase: 13 (40.6%) decrease: 17 (53.1%) same: 2 (6.3%)	**<0.001**	0.362	**<0.001**	**<0.001**	0.100	0.636
Vitamin D 1.25	increase: 10 (31.3%) decrease: 21 (65.6%) same: 1 (3.1%)	increase: 20 (62.5%) decrease: 12 (37.5%)	increase: 13 (40.6%) decrease: 18 (56.3%) same: 1 (3.1%)	increase: 13 (40.6%) decrease: 19 (59.4%)	increase: 8 (25%) decrease: 23 (71.9%) same: 1 (3.1%)	increase: 17 (53.1%) decrease: 15 (46.9%)	0.100	**0.019**	0.730	0.182	**0.001**	0.302
Prolactin	increase: 13 (40.6%) decrease: 19 (59.4%)	increase: 12 (37.5%) decrease: 20 (62.5%)	increase: 19 (59.4%) decrease: 13 (40.6%)	increase: 26 (81.3%) decrease: 6 (18.8%)	increase: 24 (75%) decrease: 8 (25%)	increase: 13 (40.6%) decrease: 19 (59.4%)	0.728	0.108	**0.027**	**<0.001**	**0.003**	0.208
Cholesterol	increase: 17 (53.1%) decrease: 13 (40.6%) same: 2 (6.3%)	increase: 8 (25%) decrease: 24 (75%)	increase: 22 (68.8%) decrease: 10 (31.3%)	increase: 21 (65.6%) decrease: 11 (34.4%)	increase: 23 (71.9%) decrease: 9 (28.1%)	increase: 15 (46.9%) decrease: 17 (53.1%)	0.587	**<0.001**	**0.029**	0.071	**0.005**	0.978
γGT	increase: 18 (56.3%) decrease: 10 (31.3%) same: 4 (12.5%)	increase: 22 (68.8%) decrease: 7 (21.9%) same: 3 (9.4%)	increase: 5 (15.6%) decrease: 24 (75%) same: 3 (9.4%)	increase: 3 (9.4%) decrease: 28 (87.5%) same: 1 (3.1%)	increase: 8 (25%) decrease: 21 (65.6%) same: 3 (9.4%)	increase: 17 (53.1%) decrease: 13 (40.6%) same: 2 (6.3%)	0.202	**0.011**	**<0.001**	**<0.001**	0.055	0.572
Creatinine	increase: 18 (56.3%) decrease: 14 (43.8%)	increase: 13 (40.6%) decrease: 19 (59.4%)	increase: 12 (37.5%) decrease: 16 (50%) same: 4 (12.5%)	increase: 19 (59.4%) decrease: 12 (37.5%) same: 1 (3.1%)	increase: 21 (65.6%) decrease: 10 (31.3%) same: 1 (3.1%)	increase: 9 (28.1%) decrease: 23 (71.9%)	0.102	0.348	0.383	0.152	**0.015**	**0.044**

Bold entries show statistically significant differences (*p* < 0.05).

## Data Availability

The raw data supporting the conclusions of this article will be made available by the corresponding author on request.

## Supplementary Materials

The following supporting information can be downloaded at https://www.mdpi.com/article/10.3390/jcm13175292/s1. Table S1: Median and Q1–Q3 range at the following time points: (a) before PRP, (b) between PRPs, and (c) one month after the second PRP. Also presented are the number and percentage of cases according to changes and the relevant *p*-value (Wilcoxon signed rank test).
