# Supplementary material for: Intraovarian Platelet-Rich Plasma Administration for Anovulatory Infertility: Preliminary Findings of a Prospective Cohort Study
Source: J Clin Med. 2024 Sep 6;13(17):5292. doi: 10.3390/jcm13175292 (PMC11396770; doi:10.3390/jcm13175292)
Supplement: Supplementary file 1 [file jcm-13-05292-s001.zip › jcm-3146176-supplementary.pdf]

Table S1. Median and Q1-Q3 range at the following time points: a) before PRP, b) between PRPs, and c) one month after the second PRP. Also presented are the number and percentage of cases according to changes and the relevant p-value (Wilcoxon signed rank test).

Measured Value	Before PRP	Between PRPs	Check 1	Check 2	Changes (before PRP and between PRPs)	Changes between PRPs and Check 1	Changes before PRP and Check 1	Changes before PRP and Check 2	Changes between PRPs and Check 2	Changes between Check 1 and Check 2	p-value (before PRP and between PRPs)	p-value (between PRPs and Check 1)	p-value (before PRP and Check 1)	p-value (before PRP and Check 2)	p-value (between PRPs and Check 2)	p-value (Check 1 and Check 2)
**AFC**	3 (2-5)	3 (2-4)	4 (3-4.5)	5 (3-7.5)	increase: 7 (21.9%), decrease: 14 (43.8%), same: 11 (34.4%)	increase: 14 (43.8%), decrease: 7 (21.9%), same: 11 (34.4%)	increase: 11 (34.4%), decrease: 16 (50%), same: 5 (15.6%)	increase: 24 (75%),, decrease: 3 (9.4%), same: 5 (15.6%)	increase: 23 (71.9%) decrease: 4 (12.5%), same: 5 (15.6%)	increase: 23 (71.9%), decrease: 6 (18.8%), same: 3 (9.4%)	0.192	**0.019**	0.329	**<0.001**	**<0.001**	**<0.001**
**FSH**	17.9 (13.15-22.15)	12.95 (9.37-15.1)	9.06 (7.58-12.4)	8.38 (7.75-10.6)	increase: 7 (21.9%), decrease: 25 (78.1%)	increase: 9 (28.1%), decrease: 23 (71.9%)	increase: 1 (3.1%) decrease: 31 (96.9%),	increase: 2 (6.3%), decrease: 30 (93.8%)	increase: 6 (18.8%), decrease: 26 (81.3%)	increase: 14 (43.8%) decrease: 18 (56.3%)	**<0.001**	**0.005**	**<0.001**	**<0.001**	**<0.001**	0.233
**LH**	15.15 (12.85-18.1)	8.08 (4.35-13.2)	6.66 (4.85-10.75)	6.88 (5.1-8.9)	increase: 6 (18.8%), decrease: 26 (81.3%)	increase: 15(46.9%), decrease: 17(53.1%)	increase: 3 (9.4%) decrease: 29 (90.6%)	increase: 4 (12.5%) decrease: 28 (87.5%)	increase: 14 (43.8%) decrease: 18 (56.3%)	increase: 13 (40.6%), decrease: 17 (53.1%), same: 2 (6.3%)	**<0.001**	0.362	**<0.001**	**<0.001**	0.100	0.636
**E2**	44 (30.92-96.9)	71.25 (45.3-138.79)	73.05 (50.27-109)	89 (53-110.16)	increase: 18 (56.3%), decrease: 13 (40.6%), same: 1 (3.1%)	increase: 16 (50%), decrease: 16 (50%)	increase: 14 (43.8%), decrease: 18 (56.3%)	increase: 11 (34.4%), decrease: 21 (65.6%)	increase: 14 (43.8%), decrease: 16 (50%), same: 2 (6.3%)	increase: 18 (56.3%), decrease: 14 (43.8%)	0.501	0.614	0.452	0.252	0.801	0.576
**Progesterone**	0.97 (0.74-1.23)	1.1 (0.56-1.67)	0.89 (0.68-2.2)	0.78 (0.65-1.65)	increase: 18 (56.3%), decrease: 14 (43.8%)	increase: 16 (50%), decrease: 16 (50%)	increase: 17 (53.1%), decrease: 14 (43.8%), same: 1 (3.1%)	increase: 16 (50%), decrease: 16 (50%)	increase: 18 (56.3%), decrease: 14 (43.8%)	increase: 16 (50%), decrease: 16 (50%)	0.188	0.608	0.381	0.826	0.311	0.222

**Vitamin D 1.25**	40 (33.1-47.2)	35.8 (28.6-42.5)	41.65 (35-46.85)	41 (33.6-53.2)	increase: 10 (31.3%), decrease: 21 (65.6%), same: 1 (3.1%)	increase: 20 (62.5%), decrease: 12 (37.5%)	increase: 13 (40.6%), decrease: 18 (56.3%), same: 1 (3.1%)	increase: 13 (40.6%), decrease: 19 (59.4%)	increase: 8(25%), decrease: 23(71.9%), same: 1(3.1%)	increase: 17 (53.1%), decrease: 15 (46.9%)	0.100	**0.019**	0.730	0.182	**0.001**	0.302
**Vitamin B12**	430.5 (391.5-457.5)	415.5 (385-467)	450.5 (379-505)	466 (415.5-513.5)	increase: 15 (46.9%), decrease: 17 (53.1%)	increase: 18 (56.3%), decrease: 12 (37.5%), same: 2 (6.3%)	increase: 12 (37.5%), decrease: 19 (59.4%), same: 1 (3.1%)	increase: 10 (31.3%), decrease: 22 (68.8%)	increase: 14 (43.8%), decrease: 18 (56.3%)	increase: 14 (43.8%), decrease: 18 (56.3%)	0.564	0.122	0.100	0.136	0.147	0.956
**Prolactin**	8.23 (5.25-11.3)	7.77 (6.38-11)	6.85 (5.4-9.68)	6.43 (4.5-8.74)	increase: 13 (40.6%), decrease: 19 (59.4%)	increase: 12 (37.5%), decrease: 20 (62.5%)	increase: 19 (59.4%), decrease: 13 (40.6%)	increase: 26 (81.3%), decrease: 6 (18.8%)	increase: 24 (75%), decrease: 8 (25%)	increase: 13 (40.6%), decrease: 19 (59.4%)	0.728	0.108	**0.027**	**<0.001**	**0.003**	0.208
**Testosterone**	23.1 (19.25-32.3)	27.58 (20.99-31.2)	23.57 (18.5-30.1)	23.3 (20.19-32.1)	increase: 19 (59.4%), decrease: 13 (40.6%)	increase: 15 (46.9%), decrease: 17 (53.1%)	increase: 16 (50%), decrease: 16 (50%)	increase: 14 (43.8%), decrease: 18 (56.3%)	increase: 16 (50%), decrease: 16 (50%)	increase: 15 (46.9%), decrease: 16 (50%), same: 1 (3.1%)	0.486	0.812	0.687	0.634	0.798	0.916
**Cortisone**	13.65 (8.9-26.8)	10.15 (6.78-18.05)	11.17 (9.4-21)	11.3 (8.9-14.45)	increase: 10 (31.3%), decrease: 19 (59.4%), same: 3 (9.4%)	increase: 19 (59.4%), decrease: 13 (40.6%)	increase: 18 (56.3%), decrease: 14 (43.8%)	increase: 18 (56.3%), decrease: 14 (43.8%)	increase: 12 (37.5%), decrease: 20 (62.5%)	increase: 15 (46.9%), decrease: 17 (53.1%)	0.051	0.241	0.244	0.113	0.614	0.205
**Cholesterol**	166 (132-201)	166.5 (156-194)	142.5 (107-156)	133 (100.5-169.5)	increase: 17 (53.1%), decrease: 13 (40.6%), same: 2 (6.3%)	increase: 8 (25%), decrease: 24 (75%)	increase: 22 (68.8%), decrease: 10 (31.3%)	increase: 21 (65.6%), decrease: 11 (34.4%)	increase: 23 (71.9%), decrease: 9 (28.1%)	increase: 15 (46.9%), decrease: 17 (53.1%)	0.587	**<0.001**	**0.029**	0.071	**0.005**	0.978

**HDL**	60.5 (53.5-71)	65.5 (57.5-76.5)	66 (57.5-72.5)	60 (48.5-69)	increase: 21 (65.6%), decrease: 11 (34.4%)	increase: 16 (50%), decrease: 16 (50%)	increase: 14 (43.8%), decrease: 17 (53.1%), same: 1 (3.1%)	increase: 16 (50%), decrease: 16 (50%)	increase: 23 (71.9%), decrease: 9 (28.1%)	increase: 9 (28.1%), decrease: 23 (71.9%)	0.070	0.582	0.262	0.362	**0.030**	**0.028**
**Trg**	81 (67-105.5)	88.5 (50-100)	73.5 (45.5-107.5)	68 (57.5-88)	increase: 13 (40.6%), decrease: 19 (59.4%)	increase: 15 (46.9%), decrease: 17 (53.1%)	increase: 25 (78.1%), decrease: 6 (18.8%), same: 1 (3.1%)	increase: 23 (71.9%), decrease: 9 (28.1%)	increase: 20 (62.5%), decrease: 12 (37.5%)	increase: 12 (37.5%), decrease: 20 (62.5%)	0.147	0.595	**0.018**	**0.001**	0.056	0.194
**γGT**	18 (13.5-21.5)	17.5 (13.5-26)	22 (17-30)	25 (21-29.5)	increase: 18 (56.3%), decrease: 10 (31.3%), same: 4 (12.5%)	increase: 22 (68.8%), decrease: 7 (21.9%), same: 3 (9.4%)	increase: 5 (15.6%), decrease: 24 (75%), same: 3 (9.4%)	increase: 3 (9.4%), decrease: 28 (87.5%), same: 1 (3.1%)	increase: 8 (25%), decrease: 21 (65.6%), same: 3 (9.4%)	increase: 17 (53.1%), decrease: 13 (40.6%), same: 2 (6.3%)	0.202	**0.011**	**<0.001**	**<0.001**	0.055	0.572
**Creatinine**	0.73 (0.58-0.99)	0.83 (0.56-1.16)	0.88 (0.63-0.99)	0.65 (0.55-0.78)	increase: 18 (56.3%), decrease: 14 (43.8%)	increase: 13 (40.6%), decrease: 19 (59.4%)	increase: 12 (37.5%), decrease: 16 (50%), same: 4 (12.5%)	increase: 19 (59.4%), decrease: 12 (37.5%), same: 1 (3.1%)	increase: 21 (65.6%), decrease: 10 (31.3%), same: 1 (3.1%)	increase: 9 (28.1%), decrease: 23 (71.9%)	0.102	0.348	0.383	0.152	**0.015**	**0.044**
**Bilirubin total**	0.53 (0.4-0.61)	0.5 (0.4-0.67)	0.4 (0.3-0.75)	0.4 (0.33-0.5)	increase: 13 (40.6%), decrease: 14 (43.8%), same: 5 (15.6%)	increase: 11 (34.4%), decrease: 15 (46.9%), same: 6 (18.8%)	increase: 16 (50%), decrease: 13 (40.6%), same: 3 (9.4%)	increase: 22 (68.8%), decrease: 8 (25%), same: 2 (6.3%)	increase: 20 (62.5%), decrease: 9 (28.1%), same: 3 (9.4%)	increase: 13 (40.6%), decrease: 16 (50%), same: 3 (9.4%)	0.788	0.701	0.696	**0.023**	0.245	0.378
**Bilirubin direct**	0.1 (0.1-0.2)	0.1 (0.1-0.2)	0.1 (0.1-0.2)	0.1 (0.1-0.2)	increase: 7 (21.9%), decrease: 10 (31.3%), same: 15 (46.9%)	increase: 10 (31.3%), decrease: 8 (25%), same: 14 (43.8%)	increase: 10 (31.3%), decrease: 8 (25%), same: 14 (43.8%)	increase: 11 (34.4%), decrease: 8 (25%), same: 13 (40.6%)	increase: 10 (31.3%), decrease: 9 (28.1%), same: 13 (40.6%)	increase: 10 (31.3%), decrease: 10 (31.3%), same: 12 (37.5%)	0.397	0.557	0.649	0.944	0.442	0.540

**17-OH-Progesterone**	0.69 (0.53-1.22)	0.72 (0.61-1.5)	0.71 (0.53-1.4)	0.74 (0.55-1.34)	increase: 21 (65.6%), decrease: 11 (34.4%)	increase: 16 (50%), decrease: 16 (50%)	increase: 13 (40.6%), decrease: 17 (53.1%), same: 2 (6.3%)	increase: 18 (56.3%), decrease: 13 (40.6%), same: 1 (3.1%)	increase: 19 (59.4%), decrease: 11 (34.4%), same: 2 (6.3%)	increase: 12 (37.5%), decrease: 20 (62.5%)	0.124	0.730	0.357	0.759	0.402	0.139
**TSH**	1.58 (1.05-2.1)	1.7 (1.12-2.2)	1.77 (1.56-1.9)	1.99 (1.65-2.15)	increase: 15 (46.9%), decrease: 15 (46.9%), same: 2 (6.3%)	increase: 15 (46.9%), decrease: 17 (53.1%)	increase: 17 (53.1%), decrease: 15 (46.9%)	increase: 13 (40.6%), decrease: 19 (59.4%)	increase: 14 (43.8%), decrease: 18 (56.3%)	increase: 22 (68.8%), decrease: 10 (31.3%)	0.552	0.654	0.627	0.110	0.756	0.083
**T3**	1.5 (1.21-1.62)	1.62 (1.23-2)	1.65 (1.45-2.05)	1.44 (1.21-1.77)	increase: 17 (53.1%), decrease: 15 (46.9%),	increase: 16 (50%), decrease: 15 (46.9%), same: 1 (3.1%)	increase: 14 (43.8%), decrease: 17 (53.1%), same: 1 (3.1%)	increase: 20 (62.5%), decrease: 12 (37.5%)	increase: 20 (62.5%), decrease: 11 (34.4%), same: 1 (3.1%)	increase: 11 (34.4%), decrease: 21 (65.6%)	0.707	0.402	0.158	0.533	0.133	**0.002**
**fT4**	1.6 (1.3-1.8)	1.8 (0.9-2.0)	1.1 (1.05-1.65)	1.12 (0.9-1.72)	increase: 13 (40.6%), decrease: 19 (59.4%)	increase: 15 (46.9%), decrease: 17 (53.1%)	increase: 11 (34.4%), decrease: 8 (25%), same: 13 (40.6%)	increase: 16 (50%), decrease: 13 (40.6%), same: 3 (9.4%)	increase: 15 (46.9%), decrease: 17 (53.1%)	increase: 11 (34.4%), decrease: 21 (65.6%)	0.740	0.307	0.572	0.608	0.502	0.402

Bold entries show statistically significant differences ($p<0.05$).
